# Long term follow up of congenital thrombotic thrombocytopenic purpura (Upshaw-Schulman syndrome) on hemodialysis for 19 years: a case report

**DOI:** 10.1186/1471-2369-14-156

**Published:** 2013-07-20

**Authors:** Koki Mise, Yoshifumi Ubara, Masanori Matsumoto, Keiichi Sumida, Rikako Hiramatsu, Eiko Hasegawa, Masayuki Yamanouchi, Noriko Hayami, Tatsuya Suwabe, Junichi Hoshino, Naoki Sawa, Kenichi Ohashi, Koichi Kokame, Toshiyuki Miyata, Yoshihiro Fujimura, Kenmei Takaichi

**Affiliations:** 1 Nephrology Center, Toranomon Hospital, Tokyo, Japan; 2 Department of Pathology, Toranomon Hospital, Tokyo, Japan; 3 Okinaka Memorial Institute for Medical Research, Toranomon Hospital, Tokyo, Japan; 4 Department of Blood Transfusion Medicine, Nara Medical University, Nara, Japan; 5 Department of Molecular Pathogenesis, National Cerebral and Cardiovascular Center, Suita, Osaka, Japan; 6 Nephrology Center, Toranomon Hospital Kajigaya, 1-3-1, Kajigaya, Takatu-ku, Kawasaki-shi, Kanagawa-ken 213-0015, Japan

**Keywords:** Congenital thrombotic thrombocytopenic purpura, ADAMTS13 (a disintegrin and metalloprotease with thrombospondin type I domain 13), Chronic hemodialysis, Complement activation, C3, Alternative pathway

## Abstract

**Background:**

Thrombotic thrombocytopenic purpura (TTP) is frequently associated with renal abnormalities, but there have been few reports about renal abnormalities in patients with hereditary TTP. In particular, little is known about the long-term prognosis of patients with childhood-onset congenital TTP.

**Case presentation:**

We report a Japanese patient with congenital TTP (Upshaw–Schulman syndrome) who was followed for 19 years after initiation of hemodialysis when he was 22 years old. At the age of 6 years, the first episode of purpura, thrombocytopenia, and proteinuria occurred without any precipitating cause. He underwent living-related donor kidney transplantation from his mother, but the graft failed after 5 months due to recurrence of TTP. Even after resection of the transplanted kidney and resumption of regular hemodialysis, TTP became refractory to infusion of fresh frozen plasma (FFP). Therefore, splenectomy was performed and his disease remained in remission for 10 years. However, TTP recurred at the age of 39 years. Plasma activity of ADAMTS13 (a disintegrin and metalloprotease with thrombospondin type I domain 13) was less than 3%, while ADAMTS13 inhibitor was not detected (< 0.5 Bethesda units/mL). The patient died suddenly after hemodialysis at the age of 41 years. Subsequent genetic analysis of this patient and his parents revealed two different heterozygous mutations of ADAMTS13, including a missense mutation in exon 26 (c.T3650C causing p.I1217T) inherited from his father and a missense mutation in exon 21 (c.G2723A causing p.C908Y) inherited from his mother. The former mutation has not been detected before in Japan, while the latter mutation is common in Japan. A retrospective review showed that serum C3 levels were consistently low while C4 levels were normal during follow-up, and C3 decreased much further during each episode of TTP.

**Conclusion:**

Congenital TTP was diagnosed from the clinical, biochemical, and genetic findings. Infusion of FFP controlled each thrombotic episode, but the effect was limited and of short duration. Review of the complement profile in this patient suggested that a persistently low serum C3 level might be associated with refractory TTP and a worse renal prognosis.

## Background

Thrombotic thrombocytopenic purpura (TTP) is a rare disorder characterized by thrombocytopenia and microangiopathic hemolytic anemia. Congenital TTP has been reported to be associated with severe deficiency of the plasma activity of ADAMTS13 (a disintegrin and metalloprotease with thrombospondin type I domain 13), which is reduced to <5% of normal by mutation of the ADAMTS13 gene, and this is known as the Upshaw–Schulman syndrome (USS) [1,2]. Deficiency of ADAMTS13 activity can also be caused by inhibitory antibodies targeting ADAMTS13, leading to acquired TTP. ADAMTS13 is a metalloproteinase that specifically cleaves multimeric von Willebrand factor (VWF) [2], while VWF is a large glycoprotein that is essential for platelet adhesion and aggregation under high shear stress conditions [3]. ADAMTS13 is mainly synthesized in the liver by stellate cells [4,5]. In addition, it is expressed by the podocytes and endothelium of the renal glomeruli, where podocyte-derived ADAMTS13 might have a local protective effect in the high shear stress glomerular microcirculation [6].

TTP is often associated with renal abnormalities and there have been some reports about such abnormalities in TTP patients, but few about hereditary TTP. In particular, there is little information about the long-term prognosis of patients with childhood-onset congenital TTP [7]. Here, we report a Japanese man with congenital TTP confirmed by genetic analysis, who was followed up for 19 years after initiation of hemodialysis.

## Case presentation

**Figure 1:**
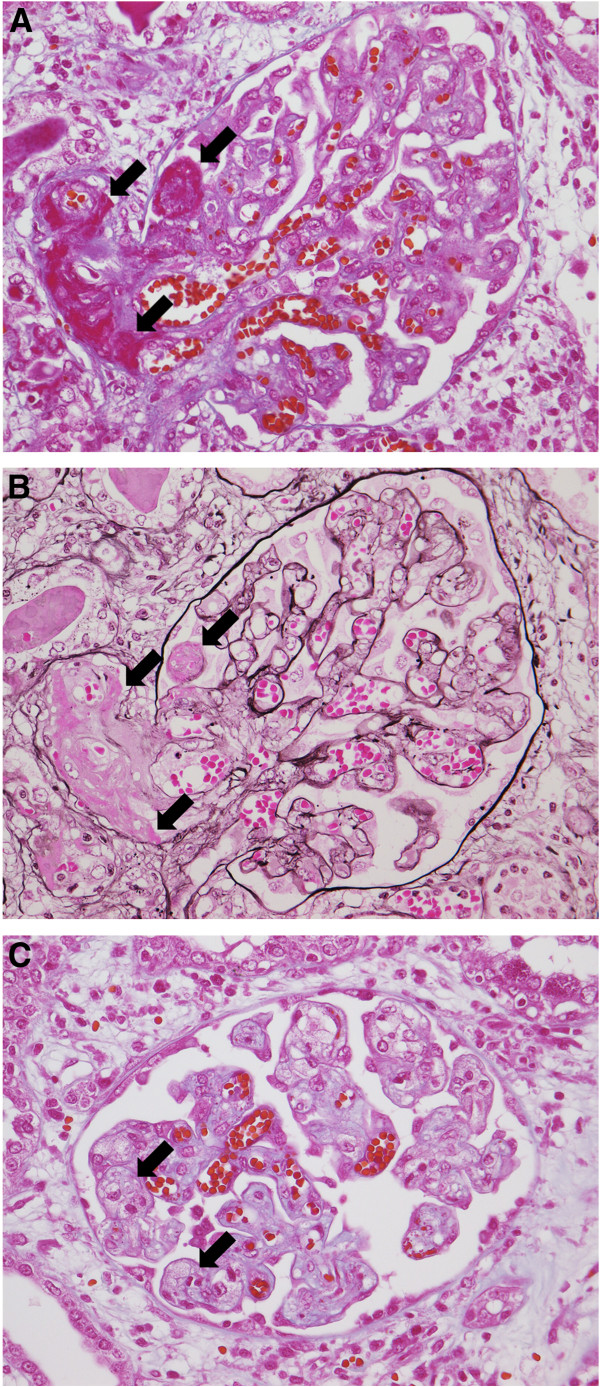
**Renal histopathological findings. A**, **B**: Fibrin thrombi in small arteries (arrows) and a glomerulus containing numerous red blood cells **(A**: Heidenhain's azan trichrome stain, **B**: Periodic acid methenamine silver stain × 400**)**. **C**: Endothelial cell swelling (arrows) (Heidenhain's azan trichrome stain × 400).

A 22-year-old man was admitted to our hospital for renal transplantation. He was the third of five children of non-consanguineous parents. There was no history of severe neonatal jaundice. Purpura of the lower extremities, thrombocytopenia, and proteinuria occurred without any precipitating cause at the age of 6 years, and hemolytic uremic syndrome (HUS) was diagnosed. This episode subsided spontaneously without treatment, but there were repeated recurrences and his renal function deteriorated gradually. In 1990, at the age of 22 years, hemodialysis was started for end-stage renal disease (ESRD) along with the occurrence of cerebral infarction. After 4 months, living-related kidney transplantation was performed with his mother as the donor. Immunosuppressive therapy included prednisolone (70 mg daily), cyclosporine (420 mg daily), antilymphocyte globulin (1 g daily), and azathioprine (100 mg daily). At 7 days after surgery, he developed thrombocytopenia (23.1 to 1.8 × 10^4^/μL) and hemolytic anemia (Hb: 10.3 to 8.2 g/dL), along with an increase of serum creatinine (1.1 to 2.1 mg/dL), lactate dehydrogenase (LDH: 208 to 785 IU), and total bilirubin (0.4 to 2.2 mg/dL). Haptoglobulin was decreased to 3.4 mg/dL. Serum levels of C3 and C4 were also decreased (C3: 63.0 to 51.7 mg/dL, normal range; 83 to 177 mg/dL, C4: 34.4 to 22.9, normal range; 15 to 45 mg/dL). Activation of HUS was suspected to have been caused by cyclosporine, so it was switched to deoxyspergualin (200 mg daily). After methylprednisolone pulse therapy (500 mg/day for 3 days) and infusion of fresh frozen plasma (FFP) (800 mL × 5 days), HUS subsided temporarily. However, there was frequent relapse of HUS, so azathioprine was changed to mizoribine and muromonab-CD3 was administered. Plasma exchange or infusion FFP was effective for terminating each episode of HUS. After 50 days, cerebral hemorrhage occurred, followed by gastrointestinal bleeding at 90 days. Then HUS recurred with thrombocytopenia and hemolytic anemia, which was refractory to plasma exchange or infusion of FFP, and his renal function deteriorated gradually. In May 1991, removal of the kidney graft was performed and hemodialysis was restarted. Examination of the resected kidney showed thrombi, endothelial cell swelling, and numerous red blood cells in the glomeruli and small arteries (Figure 1). After nephrectomy, jejunal bleeding was treated by transcatheter arterial embolization of an arteriovenous malformation in the superior mesenteric artery territory.

Even after hemodialysis was resumed, transient ischemic attacks and cerebral infarction occurred every time his platelet count decreased spontaneously, subsiding in response to infusion of FFP. However, TTP became refractory to FFP in 1998. Because indium platelet scintigraphy showed high uptake in the spleen and his platelets had a short lifespan (1.76 days), splenectomy was performed in order to prevent excessive platelet destruction. Thereafter, thrombotic episodes requiring the infusion of FFP did not occur for 10 years until 2008. During this remission period, the serum level of C3 was always lower than normal and serum C4 was normal, while the C3 level decreased much further with each episode of TTP. When cerebral infarction with thrombocytopenia occurred again at the age of 39 years, plasma ADAMTS13 activity was less than 5% of normal, as measured by the FRETS-VWF73 assay [8], while ADAMTS13 inhibitor was negative (<0.5 Bethesda units/mL) [9]. USS was diagnosed because he had severe deficiency of ADAMTS13 activity without any detectable inhibitor in conjunction with appropriate clinical criteria. Although the thrombotic episodes subsided following infusion of FFP, he died suddenly after hemodialysis in 2010 at the age of 41 years. After the patient’s death, we measured plasma ADAMTS13 activity and inhibitor in his parents using a chromogenic ELISA [10]. Both of them had ADAMTS13 activity around 30% of normal and the inhibitor was negative.

### Genetic analysis

After obtaining consent from his parents, genetic analysis of the patient and parents was performed with the approval of the Ethics Committees of Nara Medical University, the National Cerebral and Cardiovascular Center, and Toranomon Hosipital. Genetic analysis of the patient was carried out at the National Cerebral and Cardiovascular Center using DNA extracted from the resected spleen. For his parents, analysis was performed at the Department of Blood Transfusion Medicine of Nara Medical University.

**Figure 2:**
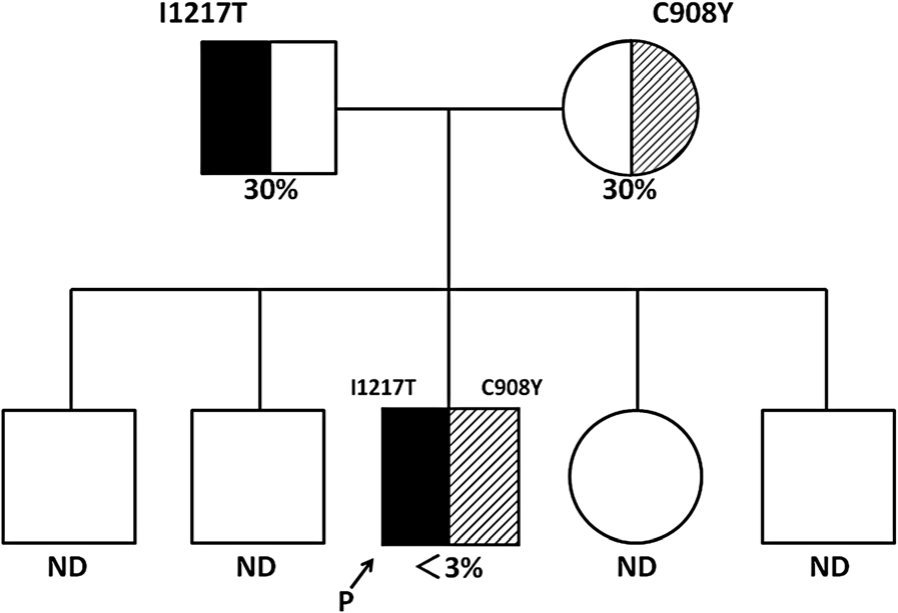
**Pedigree of the index patient with genetic haplotypes and plasma activity of ADAMTS13 (a disintegrin and metalloprotease with thrombospondin type I domain 13).** Squares represent males and circles represent females. Plasma ADAMTS13 activity (%) is shown under the circles and squares. Mutations of the ADAMTS13 gene are shown as one-letter amino acid abbreviations numbered from the initial Met codon. The arrow indicates the index patient. The mother and father of the index patient are both asymptomatic carriers. Abbreviations P: patient, ND: not determined.

It was demonstrated that the patient had compound heterozygous mutations of ADAMTS13, comprising a missense mutation in exon 26 (c.T3650C causing p.I1217T) that was inherited from his father and a missense mutation in exon 21 (c.G2723A causing p.C908Y) inherited from his mother. A diagnosis of congenital TTP (USS) was confirmed by these findings (Figure 2).

### Discussion

It is widely recognized that TTP is associated with renal abnormalities, with renal failure occurring secondary to damage caused by microthrombi that develop because of decreased plasma ADAMTS13 activity. The common renal manifestations of TTP are proteinuria and hematuria. Acute renal failure (ARF) affects 11% of patients with severe congenital TTP and often recurs with exacerbation of this disease [7]. Although ARF requiring dialysis was reported to be less frequent (0–9.7%) in four series of patients with acquired TTP [11-13], the percentage of patients with congenital TTP who need regular dialysis is unclear. Tsai et al. [7] reported that five out of nine patients with USS progressed to ESRD requiring dialysis, and three of them had episodes of ARF. Therefore, repeated episodes of ARF may be associated with progression to ESRD.

Because infusion of plasma is effective for acute exacerbation of congenital TTP, plasma exchange is the standard treatment. In patients with relapsing and/or refractory TTP, splenectomy can be effective. The mechanism is assumed to be that splenectomy decreases autoantibody production by removing a large reservoir of B lymphocytes [14], which is a reasonable explanation for patients with acquired TTP and elevated levels of ADAMTS13 inhibitor. However, Snider et al. [15] reported a patient with relapsing and refractory congenital TTP who remained in complete clinical remission for 4 years after splenectomy. In our patient, remission of TTP persisted for 10 years after splenectomy, but the effect was limited. The mechanism by which splenectomy improves congenital TTP is unknown, although it is possible that a state like idiopathic thrombocytopenia purpura (ITP) might have coexisted with TTP in our patient because his short platelet lifespan was compatible with ITP. Since TTP remained in remission for 10 years after splenectomy without the need for FFP, this case shows that splenectomy can be a useful option for relapsing/refractory congenital TTP. There has only been one previous case report of renal transplantation for chronic renal failure in a patient with congenital TTP, and the graft showed early failure due to disease recurrence [16]. In our case, the graft also failed due to chronic relapsing TTP only 5 months after transplantation. Therefore, renal transplantation may not a feasible option for ESRD in patients with congenital TTP.

Several mutations of the ADAMTS13 gene have been reported in congenital TTP. It is thought that specific ADAMTS13 mutations are more common among certain ethnicities [17]. Fujimura et al. [17] evaluated 43 USS patients in Japan and found ADAMTS13 mutations that were specific to Japanese individuals with congenital TTP. The present patient had p.C908Y with maternal inheritance, which is one of the common ADAMTS13 mutations found in Japanese patients [17]. However, the patient also had p.I1271T (inherited from his father) and this has not been reported before in Japanese patients, was although it is consistent with the missense mutation reported by Park et al. [18] in a Korean patient who had congenital TTP complicating moyamoya disease. Fujimura et al. [17] reported that two out of 43 patients with congenital TTP progressed to ESRD requiring dialysis. One of them was homozygous for c.414 + 1G > A, while the other was heterozygous for c.1885delT (paternal inheritance) and p.C908Y (maternal inheritance). However, these mutations were also detected in some of their TTP patients without progression to dialysis. In fact, five of the 43 patients had the p.C908Y mutation that was detected in our case, but only one of them progressed to dialysis during follow-up. Therefore, as Tsai et al. [7] concluded, the relation between ADAMTS13 mutation and the renal prognosis remains uncertain [17].

With regard to the occurrence of renal impairment in this patient, it may be important to focus on the complement system. Ruiz-Torres et al. [19] studied thrombotic microangiopathy patients with congenital ADAMTS13 deficiency and patients with ADAMTS 13 inhibitors, and they reported that four of out of six patients (66%) showed a moderate decrease of C3 in the acute phase, which was indicative of complement activation and consumption. They hypothesized that platelet microthrombi caused activation of the alternative pathway in patients with ADAMTS13 deficiency. Moreover, Noris et al. [20] reported 2 sisters who had the same compound heterozygous ADAMTS13 mutations, while one sister also had a heterozygous mutation of the gene encoding complement factor H, a plasma factor that inhibits activation of the alternative pathway. The second sister had severe disease, with renal involvement requiring chronic dialysis, and eventually died of a stroke. She had subnormal serum C3 levels and normal C4 levels. In addition, one of the four congenital TTP patients reported by Ruiz-Torres et al. had a subnormal C3 level even in remission and her serum creatinine level was 5.73 mg/dL, suggesting ESRD. Considering these reports, some patients with congenital TTP may have persistently low C3 levels that may be associated with a worse renal prognosis. The findings in our case seem to support this hypothesis. If a persistently depressed C3 level and normal C4 level, indicating selective activation of the alternative pathway, is one of the causes of severe TTP, the anti-C5 monoclonal antibody eculizumab may be an effective treatment for refractory TTP. In fact, Chapin et al. [21] reported that eculizumab was effective for refractory TTP, so use of eculizumab might have been a good treatment option in our case.

## Conclusion

We encountered a male patient with congenital TTP who remained on hemodialysis for 19 years. His ADAMTS13 gene had two mutations, which were p.I1217T (the first report of this mutation in Japan) and p.C908Y (common in Japan). Infusion of FFP was effective for controlling thrombotic episodes, but the improvement was limited and of short duration. The profile of complement components in this patient suggests an association of persistently low serum C3 level with refractory TTP and a worse renal prognosis.

## Consent

Written informed consent was obtained from the patient’s parents for the genetic analyses, as well as for publication of this case report and any accompanying images. We could not obtain written consent from the patient himself because he was already dead when we wrote this paper.

## Competing interests

The authors declare that they have no competing interests.

## Authors’ contributions

KM contributed to analyzing and interpretation of data and writing the manuscript. YU contributed to analyzing and interpretation of data and writing the manuscript. MM contributed to analyzing and interpretation of data and writing the manuscript. KS contributed to managing the patient and assessing data. RH contributed to managing the patient and assessing data. EH contributed to managing the patient and assessing data. MY contributed to managing the patient and assessing data. NH contributed to managing the patient and assessing data. TS contributed to managing the patient and assessing data. JH contributed to managing the patient and assessing data. NS contributed to managing the patient and assessing data. KO contributed to analyzing and interpretation of pathological findings. KK contributed to analyzing the ADAMTS13 gene of patient. TM contributed to analyzing the ADAMTS13 gene of patient. YF contributed to analyzing and interpretation of data and writing the manuscript. KT contributed to analyzing and interpretation of data and management the patient. All authors read and approved the final manuscript.

## Pre-publication history

The pre-publication history for this paper can be accessed here:

http://www.biomedcentral.com/1471-2369/14/156/prepub
